# Pré-Hipertensão Em Adolescentes: Um Novo Velho Problema

**DOI:** 10.36660/abc.20210702

**Published:** 2021-10-06

**Authors:** Katya Rigatto

**Affiliations:** 1 Universidade Federal de Ciências da Saúde de Porto Alegre Porto AlegreRS Brasil Universidade Federal de Ciências da Saúde de Porto Alegre, Porto Alegre, RS – Brasil

**Keywords:** Adolescentes, Hipertensão, Pré-Hipertensão, Pressão Arterial, Sedentarismo, Qualidade de Vida, Fatores de Risco, Intervenção Médica Precoce/tendências

Não há dúvida de que o risco de hipertensão é proporcional ao aumento da pressão arterial (PA) e não havendo um limiar específico, quanto maior a pressão, maior o risco. Nesse contexto, a identificação de indivíduos com risco cardiovascular aumentado na fase subclínica e assintomática é de suma importância^1^ para, no futuro, melhorar a qualidade de vida dos pacientes e reduzir os custos e encargos dos serviços de saúde.

Desde a primeira tentativa de mensuração do pulso arterial – realizada por Santorio Sanctorius (1561-1636) –^2^ até Riva-Rocci,^3^ que encerrou a busca por um método clínico simples de avaliação da PA, o controle da PA vem ganhando cada vez mais destaque entre clínicos e pesquisadores e os valores da PA considerados normais têm se tornado cada vez mais baixos.

De fato, fortes evidências demonstram que fatores de risco para doenças cardiovasculares (DCV) podem ser observados precocemente^4^ e, em sua maioria, podem ser evitados para reduzir a ocorrência de hipertensão na infância e/ou adolescência. Entre esses riscos estão o sedentarismo e a obesidade, cuja prevenção tem sido um grande desafio nos últimos anos.^5^

Atualmente, acredita-se que 57% das crianças serão obesas até os 35 anos^6^ e a maioria das causas de obesidade e hipertensão na infância e adolescência são modificáveis. Dentre essas causas, podemos destacar a inatividade física e os transtornos alimentares devido ao acesso a alimentos não saudáveis.^7^ Portanto, justifica-se a grande preocupação em monitorar precocemente a PA e o esforço para evitar os fatores que podem levar à hipertensão.

De 2000 a 2015, o aumento da prevalência de hipertensão na infância oscilou entre 75% e 79%.^8^ Hoje em dia, a pandemia causada pelo vírus SARS-CoV-2 aumentou o sedentarismo devido ao confinamento forçado e tornou a situação ainda mais preocupante. O sedentarismo, entre outros efeitos, estimula a remodelação do microbioma intestinal, levando ao predomínio da colonização obesogênica.^9^

Portanto, há uma necessidade urgente de prevenir o risco de DCV em jovens. É necessário aprimorar o manejo da doença na infância ou adolescência para minimizar a ocorrência de hipertensão na vida adulta. Os valores de PA que classificam um indivíduo como normotenso têm diminuído significativamente nos últimos anos, chamando a atenção para a necessidade de monitoramento da PA em jovens e intervenções cada vez mais precoces.

A possibilidade de utilizar técnicas de monitoramento da PA não invasivas, indolores e de baixo custo, como a medida da variabilidade da frequência cardíaca (VFC), facilita significativamente o diagnóstico e o tratamento de estados de pré-hipertensão e hipertensão. Nesse sentido, a VFC vem ganhando destaque, pois reflete a eficiência do sistema nervoso simpático e parassimpático no controle do sistema cardiovascular.^10^

No artigo de Macêdo et al.,^11^ fica clara a importância da VFC em adolescentes com pré-hipertensão. Os autores chamam a atenção para a associação entre aumento da PA sistólica e diastólica com a redução da VFC em adolescentes. Coletivamente, a redução da VFC e o aumento da entropia de Shannon demonstraram um desequilíbrio no controle simpatovagal quando a PA sistólica estava acima de 120mmHg. As técnicas que avaliam a VFC, embora não invasivas e de baixo custo, são pouco utilizadas na clínica médica. No entanto elas têm se mostrado eficazes para o monitoramento dos sistemas simpático e parassimpático e também podem ser úteis para o monitoramento de pacientes, mesmo antes do início dos sintomas.

Por outro lado, embora dados da literatura apontem para uma associação entre PA e ancestralidade genética,^12 - 14^ os autores não encontraram essa associação entre os adolescentes. Além disso, dados da literatura indicam que o desequilíbrio na modulação autonômica provavelmente precede as alterações no endotélio vascular^12^ que é um importante componente no controle da PA.

Coletivamente, esses achados demonstram a complexidade do controle da PA e explicam, pelo menos em parte, as dificuldades de controle desse em pacientes hipertensos. Apesar dos grandes avanços nas descobertas sobre o controle da PA, mais de 400 anos nos separam de Riva-Rocci e o manejo da hipertensão continua sendo um grande desafio.

É fundamental saber a prevalência da hipertensão e da pré-hipertensão na população. Além disso, os prontuários eletrônicos de pacientes devem ser introduzidos para todos os médicos e devem ser criados de forma que o médico não possa prosseguir com a introdução de outras informações clínicas sem primeiro inserir a medida da PA. Esse simples procedimento evitaria vários problemas no futuro e facilitaria os registros epidemiológicos.

Além disso, a monitoração do sistema nervoso autônomo, através da observação da VFC, pode ser uma alternativa de baixo custo para a identificação e tratamento precoce da modulação autonômica do sistema cardiovascular. Assim, será possível fazer um diagnóstico precoce de distúrbios reflexos que contribuem para o início da hipertensão e, portanto, trabalhar com mais rigor para evitar a hipertensão de causas controláveis. É necessário medir a PA para prevenir as consequências das DCV o mais rápido possível e não apenas tratá-las quando o problema se apresenta quase sem solução.
