# Supplementary material for: Pré-Hipertensão Em Adolescentes: Um Novo Velho Problema
Source: Arq Bras Cardiol. 2021 Oct 6;117(4):655–6. [Article in Portuguese] doi: 10.36660/abc.20210702 (PMC8528357; doi:10.36660/abc.20210702)
Supplement: Translation

# Translation: Pre-Hypertension in Adolescents: A New Old Issue

**Authors:** Katya Rigatto

**Affiliations:** 1 Brazil Universidade Federal de Ciências da Saúde de Porto Alegre, Porto Alegre, RS – Brazil

**Keywords:** Adolescents, Hypertension, Prehypertension, Arterial Pressure, Sedentarism, Quality of Life, Risk Factors, Early Medical Intervention/trends

As there is no doubt that the risk of hypertension is proportional to the increase in blood pressure (BP) and has no specific threshold, the higher the pressure, the higher the risk. In this context, the identification of individuals with increased cardiovascular risk in the subclinical asymptomatic stage is of paramount importance^1^ in the future to improve patients’ quality of life and reduce the costs and burden of health services.

From the first attempt to measure the arterial pulse, performed by Santorio Sanctorius (1561 **–** 1636),^2^ to Riva-Rocci,^3^ who put an end to the search for a simple clinical method for BP assessment, BP control has gained more and more prominence among clinicians and researchers. Consequently, BP values considered to be normal have gradually become lower and lower.

In fact, strong evidence demonstrates that cardiovascular disease (CVD) risk factors can be observed early in life^4^ and, for the most part, can be avoided to reduce the occurrence of hypertension in childhood and/or adolescence. Among these risks are sedentarism and obesity, whose prevention has been a major challenge in recent years.^5^ Currently, it is believed that 57% of children will be obese by the age of 35.^6^ Importantly, most causes of obesity and hypertension in childhood and adolescence are modifiable. Among them, we can highlight physical inactivity and eating disorders due to the access to unhealthy foods.^7^ Therefore, the great concern to monitor BP early and the effort to avoid those factors that could lead to hypertension are justified.

From 2000 to 2015, the increasing prevalence of childhood hypertension fluctuated between 75% and 79%.^8^ Nowadays, the pandemic caused by the SARS-CoV-2 virus has increased sedentary lifestyles due to forced confinement and made the situation even more worrying. A sedentary lifestyle, among other effects, stimulates the remodeling of our intestinal microbiome, leading to a predominance of obesogenic colonization.^9^

Therefore, there is an urgent need to prevent the risk of CVD in young people. It is necessary to improve management of the disease in childhood or adolescence in order to minimize the occurrence of hypertension in adulthood. The BP values that classify an individual as normotensive have been significantly reduced in recent years, drawing attention to the need to monitor the BP of young people and increasingly earlier interventions.

The possibility of using non-invasive, painless and low-cost BP monitoring techniques such as heart rate variability (HRV) measurement significantly facilitates the diagnosis and management of pre-hypertension and hypertension states. In this sense, HRV has been gaining prominence. HRV reflects the efficiency of the sympathetic and parasympathetic nervous systems in controlling the cardiovascular system.^10^

In the article by Macêdo et al.,^11^ the importance of HRV in adolescents with pre-hypertension is made clear. The authors draw attention to the association between increased systolic and diastolic BP and a reduction in HRV in adolescents. Collectively, the reduction in HRV and the increase in Shannon entropy demonstrated an imbalance in sympathovagal control when the systolic BP was above 120mmHg. The techniques that assess HRV, although non-invasive and inexpensive, are hardly utilized in clinical medicine. However, they have shown to be efficient for the monitoring of sympathetic and parasympathetic systems and could also be helpful for monitoring patients, even before symptom onset.

On the other hand, although literature data point to an association between BP and genetic ancestry,^12 - 14^ the authors did not find this association among the adolescents. Furthermore, literature data indicate that the imbalance in the autonomic modulation probably precedes changes in the vascular endothelium,^12^ which is an important component of BP control.

Collectively, these findings demonstrate the complexity of BP control and explain, at least in part, the difficulties of controlling BP in hypertensive patients. Despite great advances in the discoveries regarding BP control, more than 400 years separate us from Riva-Rocci and the management of hypertension remains a major challenge.

It is critical to know the prevalence of hypertension and pre-hypertension in the population. In addition, electronic patient charts should be introduced for all clinicians. The files should be created so that the physician cannot proceed to introduce other medical information without first entering the BP measurement. This simple step would prevent several problems in the future and would facilitate epidemiological records.

Moreover, the autonomic nervous system monitoring, through the observation of HRV, can be a low-cost alternative for the identification and early-onset treatment of the cardiovascular system autonomic modulation. Thus, it will be possible to make an early diagnosis of reflex disorders that contribute to the onset of hypertension and therefore work more rigorously to prevent hypertension from controllable causes. It is necessary to measure BP to prevent the consequences of CVD as soon as possible and not just to treat them when the problem is almost beyond remedy.
